# Recent developments in nanomaterials for upgrading treatment of orthopedics diseases

**DOI:** 10.3389/fbioe.2023.1221365

**Published:** 2023-08-09

**Authors:** Jinxiang Shang, Chao Zhou, Chanyi Jiang, Xiaogang Huang, Zunyong Liu, Hengjian Zhang, Jiayi Zhao, Wenqing Liang, Bin Zeng

**Affiliations:** ^1^ Department of Orthopedics, Affiliated Hospital of Shaoxing University, Shaoxing, China; ^2^ Department of Orthopedics, Zhoushan Guanghua Hospital, Zhoushan, China; ^3^ Department of Pharmacy, Zhoushan Hospital of Traditional Chinese Medicine Affiliated to Zhejiang Chinese Medical University, Zhoushan, China; ^4^ Department of Orthopedics, Zhoushan Hospital of Traditional Chinese Medicine Affiliated to Zhejiang Chinese Medical University, Zhoushan, China

**Keywords:** nanotechnology, orthopedic, orthopaedic implant, drug delivery, nano-diagnostics

## Abstract

Nanotechnology has changed science in the last three decades. Recent applications of nanotechnology in the disciplines of medicine and biology have enhanced medical diagnostics, manufacturing, and drug delivery. The latest studies have demonstrated this modern technology’s potential for developing novel methods of disease detection and treatment, particularly in orthopedics. According to recent developments in bone tissue engineering, implantable substances, diagnostics and treatment, and surface adhesives, nanomedicine has revolutionized orthopedics. Numerous nanomaterials with distinctive chemical, physical, and biological properties have been engineered to generate innovative medication delivery methods for the local, sustained, and targeted delivery of drugs with enhanced therapeutic efficacy and minimal or no toxicity, indicating a very promising strategy for effectively controlling illnesses. Extensive study has been carried out on the applications of nanotechnology, particularly in orthopedics. Nanotechnology can revolutionize orthopedics cure, diagnosis, and research. Drug delivery precision employing nanotechnology using gold and liposome nanoparticles has shown especially encouraging results. Moreover, the delivery of drugs and biologics for osteosarcoma is actively investigated. Different kind of biosensors and nanoparticles has been used in the diagnosis of bone disorders, for example, renal osteodystrophy, Paget’s disease, and osteoporosis. The major hurdles to the commercialization of nanotechnology-based composite are eventually examined, thus helping in eliminating the limits in connection to some pre-existing biomaterials for orthopedics, important variables like implant life, quality, cure cost, and pain and relief from pain. The potential for nanotechnology in orthopedics is tremendous, and most of it looks to remain unexplored, but not without challenges. This review aims to highlight the up tp date developments in nanotechnology for boosting the treatment modalities for orthopedic ailments. Moreover, we also highlighted unmet requirements and present barriers to the practical adoption of biomimetic nanotechnology-based orthopedic treatments.

## Introduction

Nanotechnology is still relatively new in orthopedic investigation, diagnosis, and therapy. Though, nanotechnology has revolutionized orthopedic therapy research and practice in the short period that it has been researched and used. Nanotechnology provides more accurate, better bone formation, and presumably safer techniques of treating the human body, at least in terms of infection rates and the necessity for reoperations. Nanotechnology improvements have cleared the path for several innovative applications in medicine (Anjum et al., 2021), biotechnology (Dutt et al., 2023), molecular biology (Ma et al., 2021; Arnold et al., 2022), as well as research on the environment (El-Sayyad et al., 2023). Nanotechnology has been used in the pharmaceutical industry (i.e., nanomedicine) with the advancement of a variety of methods for the prevention, diagnosis, and treatment of a wide range of illnesses, comprising treatment of cancer, tissue engineering (TE) scaffolds, diagnostic imaging, and immunotherapy as well as drug distribution. Due to their ability to imitate or copy organ components of normal bone, nanomaterials offer very interesting potential for the future development of orthopedic implants (Chen, 2022). Bone transplants are necessary for orthopedics to restore irreversible injury to native healthy bones. Nanoparticles (NPs) can substantially contribute to this by providing cellular structural support (for instance, nanofunctionalized scaffolding) and affecting cell migration, proliferation, and differentiation (Wen et al., 2023a; Kunrath et al., 2023).

**FIGURE 1:**
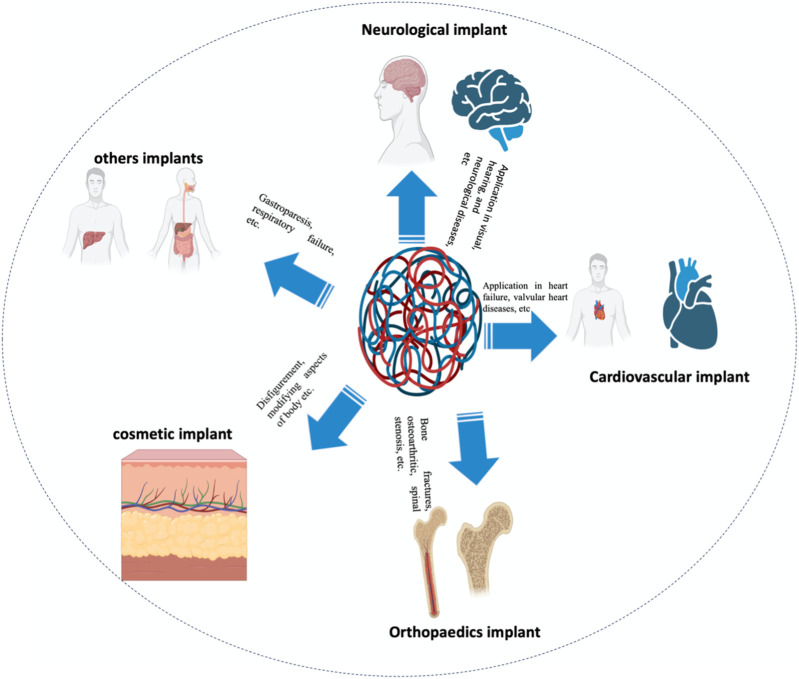
Application-related classification of bioimplants into groups’ for instance cardiac grafts, aesthetic implants, implants for the joints, neurological/sensory grafts, and many more uses is supplied.

As the population ages, variations in lifestyles (specifically those that induce and aggravate chronic ailments like cardiac and osteoarthritic disease), bioengineering technological improvements, and augmented awareness of cosmetic implants all contribute to the exponential development of the bioimplant industry. As per market investigations, the worldwide bioimplant market is anticipated to reach $115.8 billion by 2020, growing at a compound annual growth rate (CAGR) of 10.3 percent between 2014 and 2020 (Zhao R. et al., 2021). Bioimplants have emerged as a possibly revolutionary therapeutic choice for blindness, neurological disorders, orthopedic issues, heart problem, defects, and dental problems (Figure 1) (Amirtharaj Mosas et al., 2022; Davis et al., 2022). Various bioimplants, like replacement of joints, bone plates, cardiac valves, Grafts of blood vessels, grafts in the teeth, sutures, ligaments, as well as intraocular lenses, are commonly utilized to 1) repair or modify the function of damaged or deteriorating tissues, 2) modify the functioning of a physical element, 3) aid in treatment, and 4) renovate anomalies for purely cosmetic purposes (Mirzaali et al., 2022). Various engineering solutions have been documented in which conventional non-metallic/metallic substances are used to simulate the physical characteristics, chemical qualities, and tissue or organ gradient architecture (Li et al., 2023). Moreover, DNA-based implants and materials are also an attractive strategy for orthopedic applications and bone cancers (Qi et al., 2022). Though, conventional bioimplants have numerous constraints. They seldom react with tissues, are unsuited to tissue, and are not continuously tolerated by the human body (Bian et al., 2016). For instance, ceramic cranium complications are well-known. Some total ceramic hip arthroplasties have resulted in perceptible noise when the patient walks. This effect results in extremely low patient satisfaction and is a well-known reason for revision in otherwise symptom-free arthroplasty. Ceramic is also quite brittle and can fracture *in vivo*, particularly in a hard-on-hard environment, resulting in a calamitous joint failure with microscopic ceramic detritus (Szczęsny et al., 2022).

In recent years, nanotechnology’s impact on the graft industry has hastened. Particularly, NPs having biologically inspired features attract investigators to examine their potential for enhancing the functionality of conventional grafts. This review paper investigates the progress in orthopedic uses of biomaterial, from conventional (for instance, non-metallic and metallic) assets to NPs. Orthopedic therapy depends on largely precise diagnosis of therapeutic sites and effective implanting. Recent advancements in important orthopedic biomaterials, such as materials with pores, smart biomaterials, and 3D printed nanocomposite implants, as well as commercial issues, are addressed to provide a comprehensive overview of this swiftly broadening scientific field. This research offers the basis for the incorporation of nanotechnology-powered orthopedic implants within the human body.

## Implantable nanotechnology components for drug delivery

Although the major focus of nanophase medication transport systems has been on curing and avoiding infections related to grafts and artificial joints, this innovative strategy has also been demonstrated to be useful in tumor detection and therapy, as it enables a more focused attack on tumor cells and might aid in bone formation when paired with anabolic medicines to minimize osteolysis of the surfaces of prosthetic joints (Mittal et al., 2022). The advancement of injectable medicines encased in nanospheres accomplished by extending the pharmacological activity of a drug is a new and promising area of research. A few of them are discussed below.

## The function of nanotechnology-based implants in the oncology and musculoskeletal system

Savvidou et al. (2016) found that nanoscience-based orthopaedic oncology implants can substantially improve diagnostics, surmount resistance to medicines, decrease peripheral injury to healthy host tissue, and more proficiently transport medications to tumor cells. Nanomaterials have the capacity to transport ligands. By integrating specific ligands that bond to the different genes generated by cancer cells, primary and precise identification of primary and metastatic malignant bone tumours can be improved. Through the incorporation of contrast agents into NPs, it is feasible to boost the precision of targeted cancer imaging and to predict tumor survival, which could be extremely beneficial for clinical assessment and surgical planning. Cancer cells become more resistant by generating multidrug resistance (MDR) proteins on their surface, which serves as a pump, extracting the cancer drug from cells and reducing plasma amounts. NPs make it easier to create vehicles that can effectively transport cancer medicines into cells, as well as transporting specific genetic codes that can circumvent MDR proteins. Active and passive tumor cell targeting is enhanced by nanophasic drug carrier techniques. To determine the target tumor cell, drug-loaded NPs may be linked to polymers, for example, mannose and folic acid following endocytosis. As a result of their diminutive size (targeting passively) as well as the penetration of cancer cells, nanomaterials could also allow for increased medication concentrations within cancer cells. The formation of ancestor cells and haematological stem cells (HSC) utilizing zwitterionic hydrogels may facilitate the clinical application of HSC treatment (Bai et al., 2019).

Orthopedic grafts are frequently inserted in patients who have undergone resections for bone malignancy. Though, traditional substances are not meant to suppress the progression or reappearance of tumors. As a result, efforts are being developed to make implants that stimulate typical bone development while preventing tumor development. Selenium has been demonstrated to have comparable properties and nano-selenium implants have been found to avoid malignant osteoblast propagation whereas boosting bone formation at the graft-tissue interaction (Tran et al., 2010). In contrast to titanium (Ti) grafts that have not been treated, the selenium NPs increased calcium deposition, bone adhesion, bone formation, and alkaline phosphatase activity. Currently, nanosized magnesium alloy grafts with particle refinement have demonstrated antitumor properties. This substance decreased osteosarcoma cell survival and adhesion (Nayak et al., 2016).

## Implant based on nanotechnology for the cure of chondral and osseous defects

The management of segmented bone defects induced by unsuccessful fixations, trauma, or arthroplasty is extremely challenging. Present methods for correcting these issues, such as substitution of metal matrices and auto/allografts have disadvantages, such as inadequate availability, infection risk, and not enough characteristic of scaffolding, which restricts the degree of integration (Wu et al., 2022). Nanostructured nanomaterials are excellent because bone cells can colonize them (Andreacchio et al., 2018). The degree of interaction between the host tissues and biomaterial determines the best bone integration scaffold. The optimal structures must be biodegradable and function as extracellular matrices upon which cells can interact, proliferate, and distinguish into normal tissues.

Nanomaterial polymers can provide structural support as well as optimal pore sizes for cell movement and activity, and functioning as a cell migration medium and activity. In addition, they can give biochemical indicators for tissue transformation through the incorporation of chemokines and growth factors, and mechanical assistance by supplying peptide sequences that attach to receptors and stimulate intracellular pathways of signaling (Habibzadeh et al., 2022). These properties of NPs make them suitable for curing significant bone deformities. Once their biological, biochemical, structural, and templating operations have been accomplished, nanoscaffolds will deteriorate, allowing more normal healing without complications related to non-disintegrable implants and biomaterials (Roddy et al., 2018).

To remedy bone abnormalities, various nanostructured substances, both synthetic and natural, have been examined. While natural nanomaterials have high biocompatibility, their mechanical features and structural support are inadequate. In comparison, artificial substances, give greater structural support but are not biocompatible. NPs that have been synthesized, such as Bellucci et al. (2016) as well as a polymeric matrix, are currently suggested as scaffolding materials for the treatment of skeletal abnormalities due to their capacity to provide increased structural strength. External growth factor therapy like bone sialoproteins (BSP) and bone morphogenic proteins (BMP) could increase the capability of these nanostructured biomaterial to achieve efficient bone integration. Two natural polymers, gelatin, and fibrin, have been employed to restore lesions of bone in non-weight-bearing locations, for example, cranial abnormalities.

Because of the more complex structure of cartilage, It is more difficult to treat cartilaginous anomalies via artificial or biological frameworks. Due to their high biocompatibility, penetration of cells, neovascularization, and recyclability, biological protein scaffolding, for example, collagen and polysaccharides scaffolds, chondroitin sulphate, agarose, HA, and chitosan are suggested for cartilage healing (Vasita and Katti, 2006). Even though it is immunogenic, Type I collagen scaffolds are the most widely used. It has been demonstrated that MSCs found in acid-treated collagen polymers generate hyaline-like cartilage in individuals with chondral deformities. Gelatin is a substitute for collagen that has been denatured and does not result in immunoreactivity or transmission of disease.

Provided that the majority of lesion cartilage is amenable to less invasive surgical procedures, the availability of injected scaffolding is crucial. Hydrogels are injectable nanoscale systems composed of polymers containing collagen or gelatin that can solidify and adhere to the contour of the fault following implantation. When equipped with chondrocytes and administered, Hydrogels have been proven to generate an ECM that resembles cartilage with a continual increase in mechanical properties due to the constant development of a glycosaminoglycan-rich matrix.

Utilizing nanofibers to make chondrogenic or osteogenic frameworks have resulted in various advantages, comprising of improved cell adhesion, movement, and propagation. The scaffolds made of nanotubes had the highest amount of type II collagen, a greater ability to absorb proteins from human blood, and a substantial increase in the amount of expression of cartilage-specific genes and proteins, for instance, collagen IX and II (D’Antimo et al., 2017) recognized cartilage and bone TE defects as one of the most significant applications of nanotechnology and interrelated investigations in orthopedics.

## Orthopaedic biomaterial classification and challenges

**FIGURE 2:**
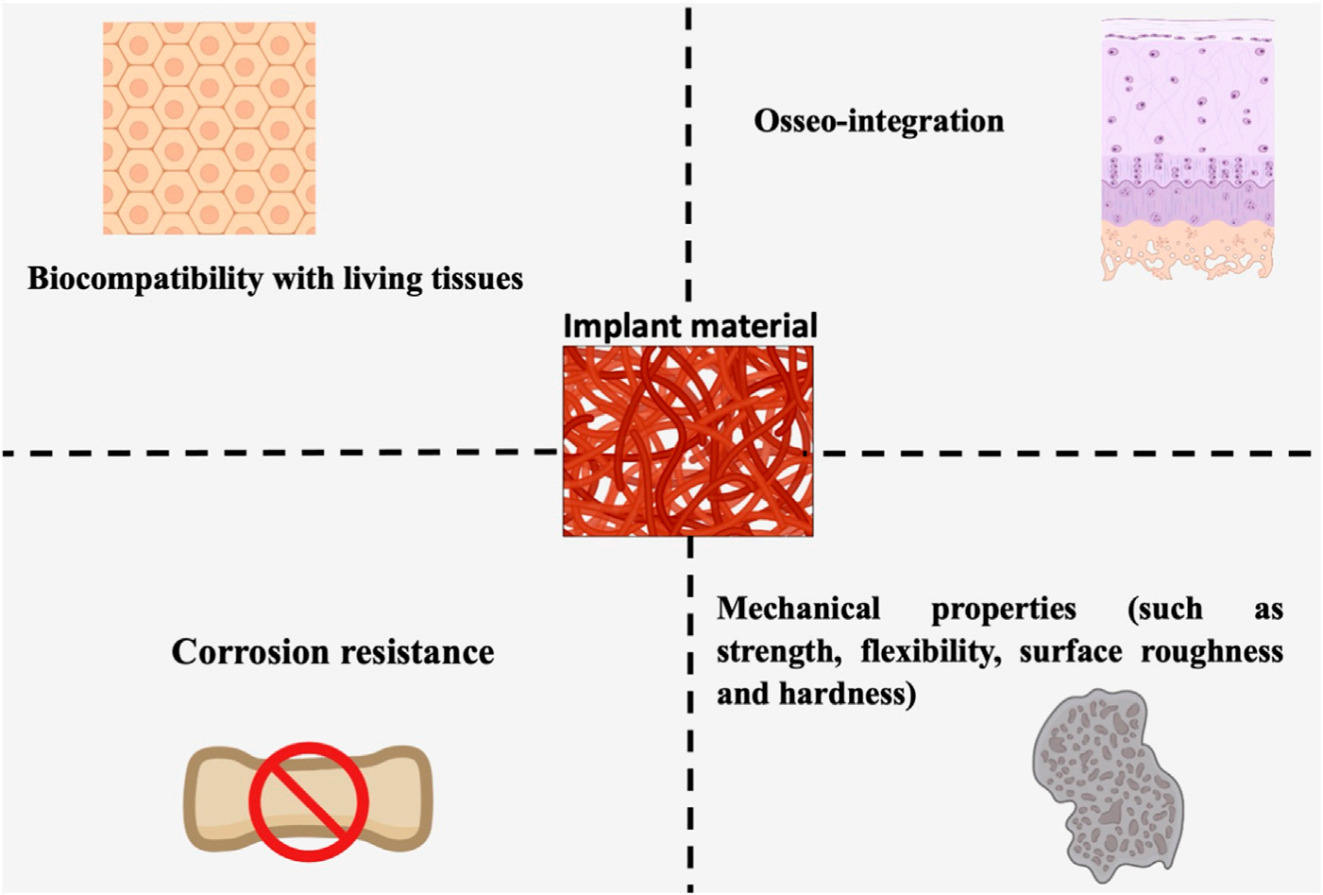
Living tissue biocompatibility, resistance to corrosion, osseointegration, and mechanical features are all essential design factors for orthopedic biomaterials (strength, flexibility, surface, roughness, and rigidity).

Soft tissues (ligaments, synovial membranes, skin, and fibrous tissues) and hard tissues (bone, cartilage, teeth, and nails), which can or could not contain mineral components, are the two major categories of biological tissues. Investigators have developed novel techniques for modeling or duplicating organs due to the scarcity of organ donors (Carvalho et al., 2018). In response to this need, bioimplants have been devised to renovate, maintain, or improve the functioning of human tissues. Although biomaterials intended for grafting applications are identical to biomolecules such as tissue and bone, they are not similar. These artificial or natural biomaterials are designed to act correctly in biological environments. In orthopedic grafts, biomaterials are utilized to heal or modify the structural integrity of fractured bone. Several key conditions must be met by each biomaterial, for instance, suitable mechanical characteristics (for instance, accurate weight and elastic modulus), excellent biostability (corrosion protection, oxidation, and hydrolysis), high bio-inertness (non-irritant and harmless), biocompatibility, in the case of bone prosthesis (integration of the osseum), and easy operative use (Figure 2) (Mitragotri and Lahann, 2009).

Sridharan et al. (2015) have presented biological biomaterials that promote cell proliferation and tissue remodeling. Huebsch and Mooney (2009) and Achterberg et al. (2014) report that significant research attempts have been undertaken throughout history to create and control biomaterial properties to accomplish uses-particular biological reactions. By modulating the rigidity of the cell-substrate, for instance, it is possible to boost muscle cell growth. In the present day, orthopedic biomaterials can be classified into 02 main classes: traditional biopolymers and nanostructured biological materials. The traditional biomaterials can be classified widely into the following groups: 1) nonmetallic constituents (such as glasses that are amorphous, polymeric materials, carbon nanocomposite, and crystalline ceramics) and 2) metal and metal alloy. This article examines conventional biomaterials and the challenges they raise in orthopedic implants application.

## Metals and alloys

Frequently, alloys and metals are chosen for load-bearing and anteriorly-fixed orthopedic implants. These transplants are securely affixed to the bone, certifying minimal mobility between the graft and the recipient tissue and load-bearing capacity at the site of insertion. Only a few of these substances are recyclable, and hence able to perform long-term success in graft uses, such as magnesium (Mg) in load-bearing reusable orthopedic implants (Julmi et al., 2019), stainless steel surgical grade (typically 316 L) in nonpermanent grafts (e.g., hip nails and plate fracture) (Zlotnik et al., 2019), and Ti in bone and joint replacement (Kaur and Orthopaedic biomaterials applications require specific mechanical properties for 1) stabilizing or enhancing fracture strength, 2) realignment of fragments of bones, and 3) joint substitutions. During the 1860s revolutionary industrialization, when the metal industries were experiencing growth, metallic nanomaterials were first utilized (Lausmaa et al., 1990). Metallic substances play a key role in biomedical graft engineering because of their homogenous qualities (such as a high degree of toughness, resilience, and tensile strength), manufacturing simplicity, and adequate biocompatibility, which are all looked-for graft lifespan (Kaur et al., 2017; Jenko et al., 2018; Dogra et al., 2019). Ti and its Ti6Al4V alloy are widely used in bioimplants constituents because of their fatigue performance, resistance to corrosion, excellent biocompatibility, lower cobalt level, and light weight. The zwitterionic metal-chelating polymers have improved biological distribution, higher radioactivity in the blood, reduced absorption by typical tissues, and enhanced tumor absorption. Making use of the HER2 extracellular domain, the resonance of surface plasmons experiments revealed that MCP immune conjugates were shown to have high antigen binding capabilities, with the dissociation constant in the sub-nM range (Liu et al., 2015). Utilizing a fluorescein neutravidin/isothiocyanate receptor as an intermediary, conjugation to biotinylated beads was made possible by a biotin end group. To demonstrate the behavior of the material, high-resolution image mass cytometry investigations based on 115 whereas recognition was conducted. The results suggest that [In(cb-te2pa)]+ complex-based polymers may be used in the future for mass cytometry of bio-implant (Grenier et al., 2020).

**FIGURE 3:**
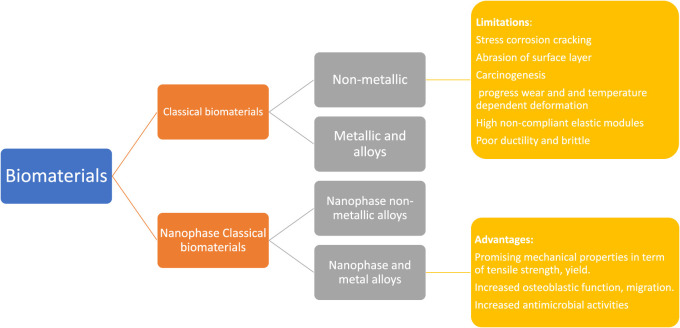
Orthopedic biomaterials are divided into 02 categories: traditional biomaterials and nanophase biomaterials. The previous is further categorized as 1) biomaterials made of metals and alloys and 2) biomaterials that are not metallic. Similarly, the later can be classified as I nanophase metallic (and alloyed) biomaterial and 3) nano-phase nonmetallic biomaterials. Traditional biomaterials have several drawbacks in implant application, containing corrosion procedure (in the case of 316 L steel substance), progressive deterioration, and thermally deformation are a few of the issues that can happen (in the case of polymers). In contrast, NPs have several attractive properties, including improved function of osteoblast and mechanical features (in terms of tensile and yield strength, etc.).

The development of an oxide coating on metal surfaces allows excellent corrosion resistance. According to Kurella and Dahotre (2005), the stability of the oxide coating on bioimplants is a major concern because it can change as a consequence of relations between the metallic surface and living tissues. The majority of metallic components disintegrate chemically and/or electrochemically in the surroundings of the human body, which consists of an oxygenated saline solution containing a salt concentration of 0.9%, a 37°C temperature, and a pH of 7.4 (Figure 3) (Manrique-Moreno et al., 2011). This oxygenated solution of saline contains, which includes the biological environment, metallic nanomaterials that have the potential to shed electrons in the solution. In such environments, these biomaterials are susceptible to corrosion, which might result in irritation and implant loosening (Li et al., 2003; Manam et al., 2017). Metallic bioimplants may fail prematurely due to the interaction of the corrosive bio-environment’s characteristics and physiological stresses. This early failure is caused by cracking due to stress corrosion of reusable metal implants, which can lead to significant material deterioration and shorten the graft’s lifespan (Choudhary et al., 2014). 316 L stainless steel implants have been demonstrated to fail because of their poor fatigue strength and/or sensitivity to distortion due to plasticity (Reclaru et al., 2001).

## Non-metallic materials

Many non-metallic substances, including polymers, crystalline ceramics, amorphous glasses, carbon composites, and carbon composites, exhibit intricate structural implantation features. These materials were not extensively used because of their inherent biocompatibility or lack of mechanical properties. Denry and Kelly (2014) state that significant efforts have been made to refine them as structural transplants. Due to their improved osseointegration and rejuvenation of bones abilities, polymeric materials are chosen for 1) Porous scaffolds in tissue TE and 2) controlled drug biocompatibility and exceptional electromechanical properties (Middleton and Tipton, 2000; Farah et al., 2016). Polymers have two advantages over metallic implants: 1) they can progressively convey stress to injured regions, enabling optimum healing of tissues; and 2) they can gradually restore tissue function without the use of catalysts or enzymes. Initially, biodegradable non-reinforced polymers are superior to reinforced steel materials in terms of tension and deformation by 36% and 54%, respectively. Fibre reinforcement can enhance the tensile strength of polymer substances. It is crucial to carefully choose the packing substance that will link the transplanted equipment to the human body before implanting devices. It must be feasible for these compounds to stop waste from moving between them. According to Athanasiou et al. (1998) and Nair and Laurencin (2007), ultra-high molecular weight polyethylene (UHMWPE), poly lactic acid (PLA), polyglycolide (PGA), polyhydroxyalkanoates (PHAs), polyvinylidine fluoride (PVDF), ultra-high molecular weight polyethylene (UHMWPE), and polyether ether ketone (PEEK) are the most frequently used polymers for packing orthopedic grafts. Advanced deterioration and temperature-dependent defect under load, which is analogous to rusting in the case of implant materials made of metal, are the main problems with polymers (Sabir et al., 2009). The most frequent issue with UHMWPe is oxidative damage during shelf life, which is preventable (for example, by using certain, appropriate connecting techniques). Nevertheless, Because of the small coefficient of friction with metal, they appear more plausible as a surface for complete joint expedients (Sobieraj and Rimnac, 2009). Table 1 summarizes the application of various nanoparticles in the field of orthopedics.

**TABLE 1:** Various nanoparticles and their application in orthopedics.

	Carrier	Cargo	Outcome	References
Liposomes	(HA)-liposomal	Dexamethasone, Diclofenac	Significantly alleviating OA pain and being biocompatible	Chang et al. (2021)
	SLN system	pDNA for integrin *β*1 overexpression	Reduced chondrocyte apoptosis and improved tissue healing	Zhao et al. (2020)
	Hollow zoledronic acid-contained nanoparticles	ZOL and Ca^2+^	A Precisely Controllable Bone-Penetrating Drug Release System Allows Localised Therapy of modulating Osteoblast-Osteoclast Interactions to Prevent Osteoporotic Fractures	Liang et al. (2023)
Micelles	Polyethylene oxide- (PEO-) and polypropylene oxide- (PPO-) based polymeric micelles	rAAV sox9	Increasing ECM components accumulation and cell survival while decreasing inflammation	Urich et al. (2020)
	tetracycline (TC)-grafted methoxy poly-(ethylene-glycol)‒poly-(D, L-lactic-co-glycolic acid) (mPEG‒PLGA) micelles (TC‒mPEG‒PLGA) with TC and mPEG‒PLGA	Astragaloside IV	The nano drug delivery system (TC‒mPEG‒PLGA) could target bone *in vitro* and *in vivo*, whereby it might be employed as a new delivery technique to improve the therapeutic benefits of medicines with osteoporotic activity	Que et al. (2022)
	PNIPAM-PMPC	Diclofenac sodium	PNIPAM-PMPC nanospheres are biocompatible and increase anabolic gene expression though decreasing articular cartilage catabolism gene expression	Zhang et al. (2020a)
	Acid-activatable polymer	Curcumin	Inhibition of tumor necrosis factor-alpha (TNF-*α*) and interleukin 1*β* (IL-1*β*). Strong anti-inflammatory and antioxidant properties	Kang et al. (2020)
Exosomes		miR-140, miR-100-5p, miR-9-5p, miR-1405p, miR-135b, and lncRNA KLF3-AS1	Lowering inflammation and increasing the synthesis of cartilage markers	Tao et al. (2017), Liu et al. (2018), Liang et al. (2020)
		pTa	This new composite scaffold can successfully induce bone regeneration in locations with significant bone defects	Yang et al. (2023)
Inorganic NPs	Membrane-disguised Fe_3_O_4_	Kartogenin	Accelerating and improving the regeneration of cartilage	Zhang et al. (2020b)
	MSNs	Gold-based nanoformulations, MnO_2_, CeO_2_	The activation of the genes ACAN and COL2a1 in chondrocytes was dramatically reduced	Famta et al. (2020), Chen et al. (2021)
	Zeolitic imidazolate framework-8	*S*-Methylisothiourea hemisulfate salt	Decreasing NO and H_2_O_2_ levels, therefore limiting the development of HIF1 and M1 macrophages and improving mitochondrial function	Zhou et al. (2019)
Polymer NPs	LbL polymer microcapsule	MnO_2_	Reducing H_2_O_2_ and safeguarding cells against oxidative stress	Marin et al. (2020)
	Poly (D,L-lactic acid)-poly (ethylene glycol)-poly (D,L-lactic acid)	BMP2	causing graft differentiation into bone and cartilage while also being destroyed without toxicity	Wu et al. (2020)
	CD-PMPC	Silica	Increasing cutaneous tissue penetration and lubrication, as well as promoting medication release	Zhao et al. (2021b)
	PLGA-PEG	4MAL, kartogenin (KGN)	Increasing the retention of IA drugs for the management of OA. enhancing the amount of sulfated glycosaminoglycans	Zerrillo et al. (2021)
	Heparin electrostatic self-assembly and ε-poly-l-lysine	Platelet lysate	The platelet lysate is dispersed equally	Tang et al. (2021)
	Cell-free fibrous HA electro spinning	SDF-1*α*, TGF-*β*3	Increased recruiting and MSC infiltration to improve cartilage tissue development	Martin et al. (2021)

## Nanotechnology used in orthopaedic implants

Nanomaterials have been investigated for their bio-implant behaviors in the past due to their bioactive nature and programmable surface features (Sobieraj and Rimnac, 2009; Waterman et al., 2011; Xu et al., 2011). Nanomaterials may be utilized in orthopedic grafts due to their ability to reduce the proportions of essential biological bone constituents. For example, nanocomposites and nanomaterial monomers have been thoroughly investigated in bone TE to improve osteointegration, promote osteoblast activity, and treat bone-related illnesses. Nanosized materials used in implants, such as nanopillars, nanotubes, nanocubes, quantum dots, nanorods, nanoflowers, and metal-organic frameworks (Bhanjana et al., 2017a; Bhanjana et al., 2017b; Kumar et al., 2018; Nehra et al., 2019), are crucial to contemplate. Numerous investigations have been made into the advantageous surface features of nanoscale components that may uphold or allow 1) many specific protein relationships, 2) enhanced osteoblast increment, and 3) enhanced osteoblast improvement as well as mobility for more effective bone progression than conventional apparatuses (Zhang, 2004; Tran et al., 2009).

### Implantation of nanomaterials

With the advent of nanotechnology, a vast array of nanophase (particle size of 100 nm) constituents, containing metals, composites, ceramics, and polymers have been created with distinctive surface features; some of these substances exhibit an enhanced ability to osseointegrate and form new bone (Zhang and Webster, 2009). According to Estrin et al. (2009), the reduction of Ti particulate size from 4,500 to 200 nm (produced by analogous channel angular forcing) increases the proliferation of cells by a factor of 20. Due to their unique atomic structure, nanophase components have a dense distribution of particle boundaries. Materials with nanocrystalline structure, polycrystalline substances having crystallites as tiny as a few nm in diameter, offer superior strength and/or stiffness but are fragile and/or plastic (Koch, 2003; Meyers et al., 2006). Noteworthy is the fact that the inelasticity of nanoscale ingredients may pose insuperable obstacles for sophisticated uses for structures. This phenomenon has historically been associated with embrittlement caused by hydrogen, storing hydrogen, and the usage of metal hydrides as hydrogen detectors (Wadell et al., 2014). For single-crystal nanoparticles, the role of lattice coherency strain and displaced nucleation in particle-size dependency of hydride formation has been investigated recently (Griessen et al., 2016).

**FIGURE 4:**
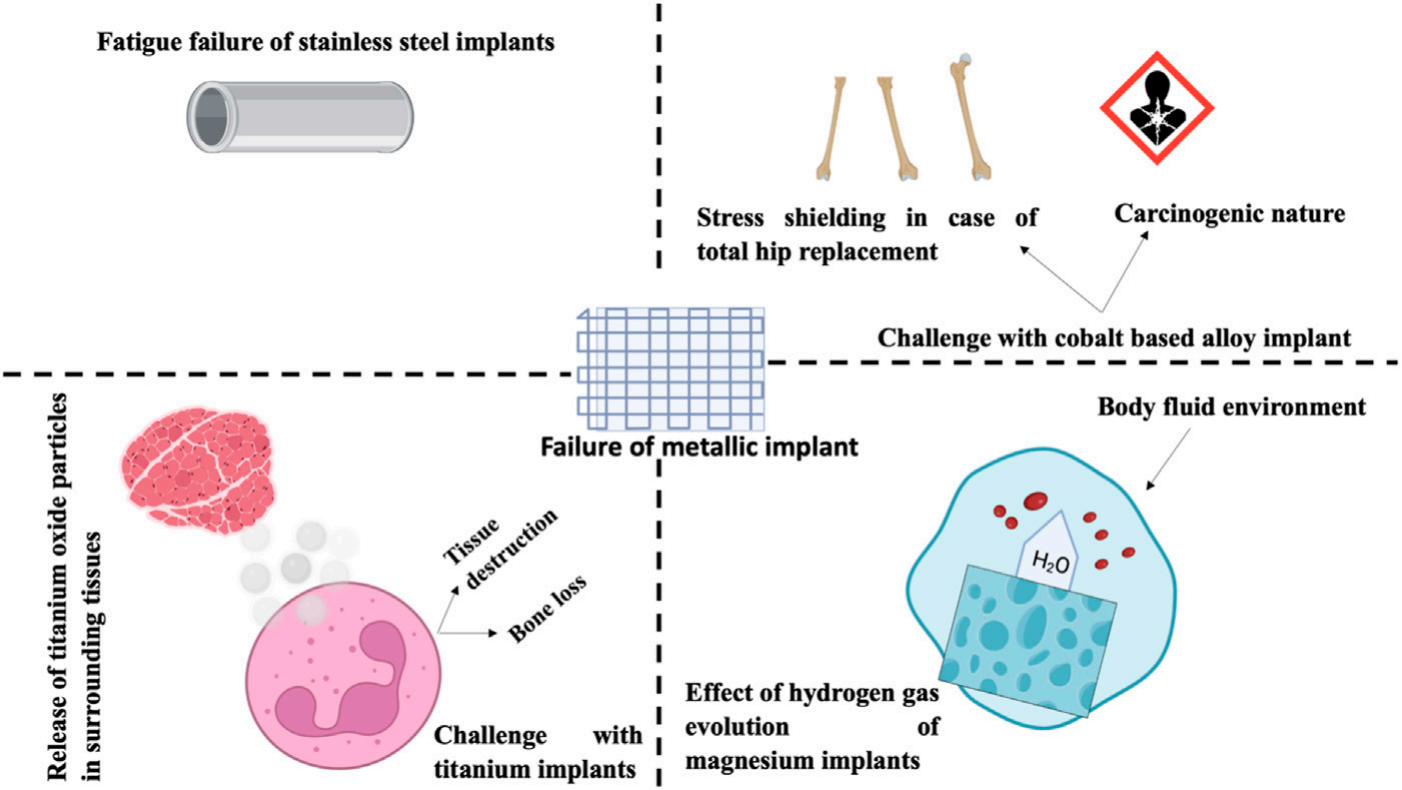
These issues are associated with conventional metal-implanted orthopedics: Failure of I stainless-steel transplant (i2) the stress resistance and carcinogenicity of cobalt alloys (i3) Titanium implants emit oxide particles into the surrounding tissue, which may result in tissue harm or loss of bone, and (i4) the effect of hydrogen gas evolution on Mg orthopedic implants.

Nanostructured materials are brittle for a variety of reasons, including their small-scale production and fundamental nature (Zhao et al., 2006). Orthopedic implants exhibited characteristic nanostructures (Webster and Ejiofor, 2004; Puckett et al., 2010; Zhou and Lee, 2011; Costa et al., 2012; Misra et al., 2013). Zhang et al. (2013) found improved mechanical features (rigidity of 31.7 GPa and Young’s modulus of 314 GPa) in NPs MgAl_2_O_4_ ceramics (grain size of 40 nm) produced by high-pressure and high-temperature sintering. Serra et al. (2013) created a nanostructured Ti6Al4V alloy by applying radical plastic defects to pure Ti. The nanostructured Ti6Al4V alloy exhibited superior mechanical properties than pure Ti, with an eventual tensile strength of 1,240 MPa versus 700 MPa. 1) yield stress of 1,200 MPa versus yield stress of 530 MPa, and 2) elongation of 12% versus 25% for pure Ti. Although the aforementioned mechanical properties, the surface roughness of nanostructured substances had a substantial impact on the function of osteoblast; Surface texture attributes for conventional Ti and 03 nanoscale constituents (Ti, CoCrMo, and Ti6Al4V alloy) were 4, 11, 9, and 356.7 nm, respectively. Utilizing a variety of nanoscale substances (such as Ti6Al4V, CoCrMo, and Ti), it has been demonstrated that osteoblast functions are enhanced in combination with diminished competitive function of cells (Webster et al., 2000; Liu J. et al., 2014) reported enhanced proliferation of osteoblasts (expressed in cells per square cm after 5 days) with all nanophase substances comprising alumina (6000), titania (8000), and HA (9000) in comparison to traditional borosilicate glass (5000). Figure 4 describes the numerous causes of the failure of metallic implants.

### Diagnostic applications

The implant function of nanotechnology in the identification of cancer is based on the capacity of nanomaterial combinations to bind to certain genetic changes, permitting cellular-scale imaging. By the addition of counterarguments to these mixtures, it is possible to observe tumor cells expressing the specific mutation (Savvidou et al., 2016).

In contrast to MRI, numerous nanotechnology-based detection instruments are transforming the field of orthopedics. In the instance of osteoporosis, diagnostic methods are essential for providing precise data quickly, affordably, and noninvasively. Before the development of nanomaterials-based methods, few reliable detection techniques were available (Garimella and Eltorai, 2017). Nanotechnology-based innovations facilitate the portable diagnosis of osteoporosis. Notably, the investigation has led to the advancement of a biochip that employs gold nanoparticles in the detection of an osteoporosis-related protein (Garimella and Eltorai, 2017) and (Garimella and Eltorai, 2017) demonstrated that this method effectively evaluates bone integrity and precisely detects and identifies bone deterioration. In addition, using fluorescent probes for identifying NPs might aid in the assessment of tumor treatment response (Hennig et al., 2015). This method can offer a more accurate estimate of the residual tumor volume rather than histopathological examination following tumor deletion (Young et al., 2012).

### Nano-diagnostics

**FIGURE 5:**
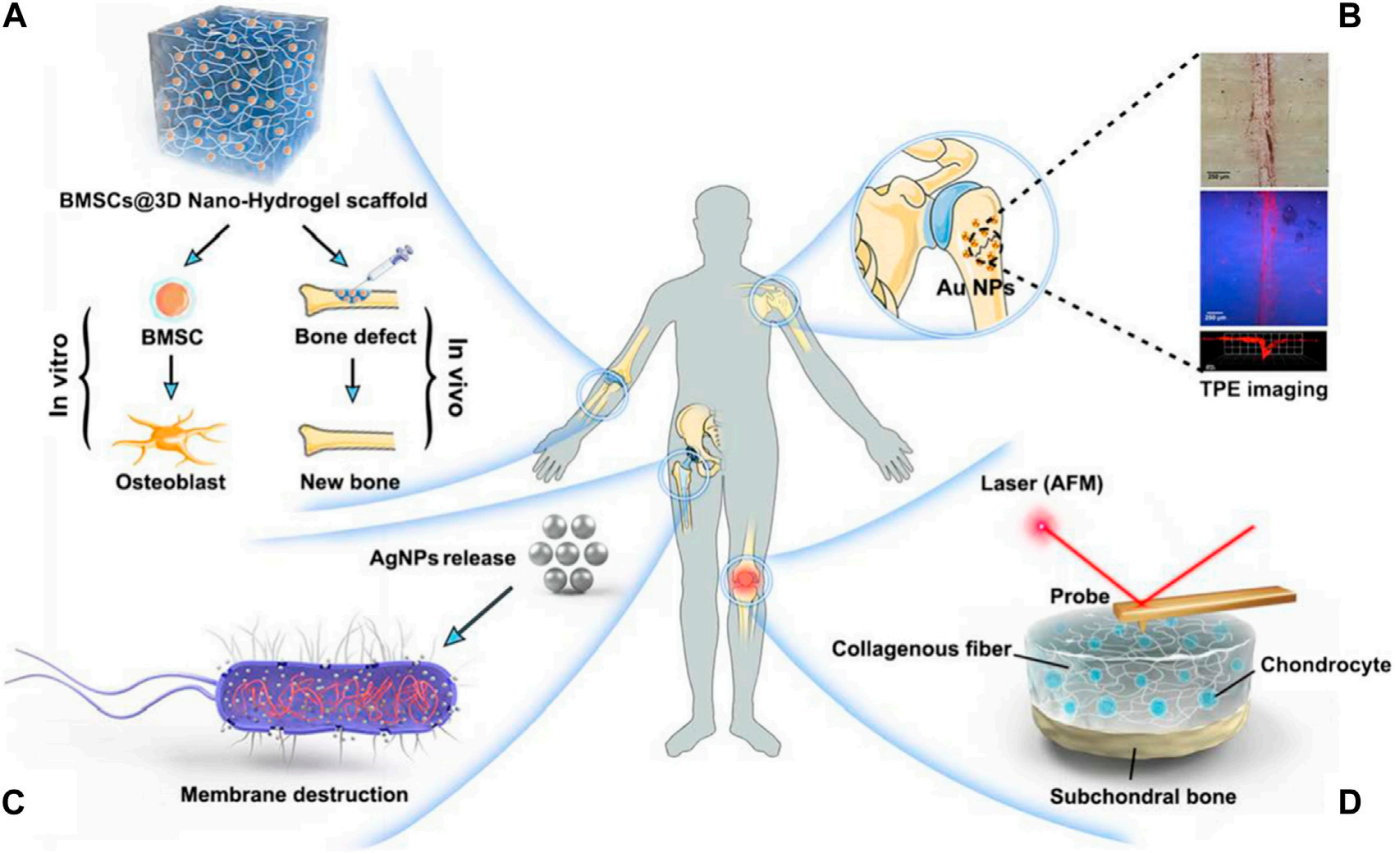
Orthopaedic nanomedicine uses. **(A)** Innovative biomimetic tissue renewal NPs **(B)** Nanoparticulate MRI contrast materials provide low-radiation, disorders of skeletal imaging at high-resolution **(C)** Design and fabrication of drug distribution devices with specific functionality utilizing nanotechnology **(D)** Usage of AFM in the initial identification and nanomechanical investigation of deteriorating joint disorders (Qiao et al., 2022).

Nano-diagnostics typically involves the use of nanostructures for labeling, tracking and detection, signal augmentation, or transformation in living beings, as well as the detection of molecules with biological activity, to accomplish rapid illness detection and point-of-care testing (POCT). The primary investigation instructions are bio-barcode assay (BCA) (Amini et al., 2019), nanoparticles (Maeda et al., 2006), nanofluidic array (biochip) (Jambari et al., 2017), nanobiosensors (Ijeomah et al., 2016), and quantum dot (QD) (Fang et al., 2021), which are used for extremely sensitive sensing, multiplex and high-throughput analysis, cell detachment and non-invasive tracing of cells, labeling fluorescent, as well as nanoprobe technologies, correspondingly. To identify biomarkers associated with inflammation like interleukin-6 (IL-6) and C-reactive protein, Borse and its colleague created a lateral flow immunoassay (LFIA) approach using fluorescent cadmium telluride QDs and a double-antibody sandwich method. When LFIA data were compared to those of the conventional enzyme-linked immunosorbent test (ELISA), it was discovered that LFIA exhibited great precision as well as sensitivity, this might help determine the condition of the grafts (Borse and Srivastava, 2019). In a separate investigation, Jin et al. created a nitric oxide (NO) non-invasive nanosensor, a real-time assessment of the progression of osteoarthritis. NO detecting molecules (4-amino-5-methylamino-2′,7′-difluorofluorescein Diaminofluorescein-FM) were encapsulated in recyclable poly (lactic-co-glycolic acid) NPs to construct this nanosensor. *In vitro* studies indicated a link between increased fluorescence intensity and changes in chondrocyte NO levels. *In vivo* tests validated its ability to determine NO concentration in the joint fluid of an OA model rodent (Jin et al., 2017). In addition, a biochip containing gold nanoparticles has been created to identify osteoproteogerin (a protein associated with osteoporosis), which may evaluate the remodeling of bones and accurately diagnose bone damage (Singh and Kim, 2012). Additionally, innovative technologies, for example, atomic force microscopy (AFM), have been employed in the investigation of the micromechanical properties of bone tissue. Hengsberger et al. demonstrated the advantages of AFM over traditional optical microscopy by combining nanoindentation and AFM methods on compact and trabecular bone tissue. 04 bone structural units (BSU) were chosen at random from desiccated bone tissue and 24 indents were examined at a maximal force of 5 mN. The findings indicate that AFM could successfully capture the surface characteristics of BSUs, accurately detect the indentation regions, and identify each BSU’s inherent mechanical characteristics, which typical optical microscopy (Hengsberger et al., 2001) was unable to achieve. Real-time dynamic and high-resolution imaging monitoring of live cells in culture (Figure 5), AFM would provide light on the relationship between the external cellular mechanical signals of chondrocytes and chondrogenesis controlled in typical and abnormal conditions (Qiao et al., 2022).

### Molecular imaging

In current years, molecular imaging has become a popular area of investigation in the field of medical imaging. Molecular imaging aims at the quantitative and qualitative investigation of biological procedures at the molecular and cellular stage utilizing live imaging technologies, thereby allowing first, *in vivo*, and targeted disease identification (Weissleder, 1999). Currently, the relatively mature technique for detecting precancerous lesions and microscopic tumors is isotope labeling imaging, which requires the administration of radioactive contrast materials with inadequate image quality, for instance, positron emission computed tomography. With the progress of nanotechnology, a great variety of MRI nanoparticulate contrast agents have become available (for example, nanoprobes made of superparamagnetic iron oxide) have been created, displace nuclear medicine chemicals employed in molecular imaging and allow for low-radiation, skeletal illnesses of high-resolution imaging. In contrast to nuclear drugs, MRI has a superior spatial resolution (up to 25–100 m) and provides multi-series imaging with instantaneous physiological and anatomical data gathering (Kozlowska et al., 2009). Surender et al. (2016), for instance, created europium-emitting surface-modified AuNPs as MRI contrast agents and showed their self-enrichment on calcium-ion-rich surfaces (microdamage to the bones caused by fatigue) for the precise marking of microcracks inside the bone. The inclusion of AuNPs may help lower the amount of contrast chemicals needed for injection, lessening the probe’s toxicity. This approach may be employed to determine the bone quality and bone mass to pinpoint the most likely fracture areas for initial targeted treatment, and furthermore the inclusion of AuNPs may minimise the amount of contrast chemicals needed for injection, minimising the toxicity of the probe.

### Bone healing with nanomedicine

**FIGURE 6:**
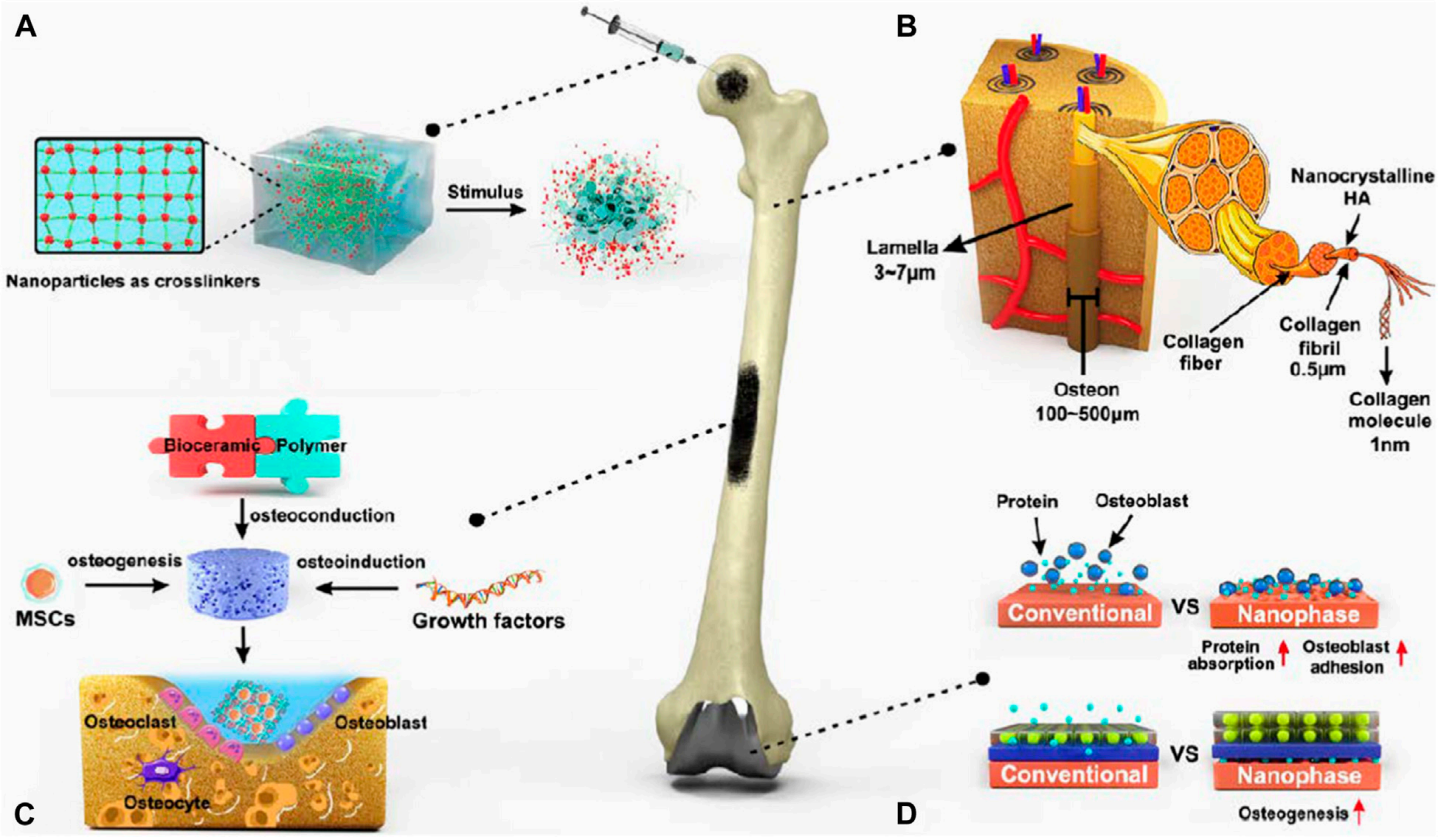
Nanotechnologies for bone healing. **(A)** The addition of NPs may significantly enhance the structural features of the hydrogel network by transmitting stimulus responsiveness while improving mechanical qualities. **(B)** The bone tissue of ECM is mostly composed of extremely organized nanocrystalline HA and collagen nanofibers. **(C)** Three critical components of bone rejuvenation using nanocomposite scaffolds as well as the cellular makeup of bone tissue. **(D)** Developing nano-surface characteristics on metallic grafts by altering their surfaces to improve protein adsorption and osteoblast adhesion, hence encouraging osteogenesis (Qiao et al., 2022).

Bone is a kind of connective tissue that is mineralized and made up of three different cell types (osteoblast, osteocyte, and osteoclast) and a biphasic extracellular matrix (the mineral-to-organic ratio is about 7:3) (Kolbasi et al., 2020). The creation of the skeleton by collagen fibers and cells (primarily COL-I) initiates bone metabolism. The deposition of minerals into the skeleton is controlled by growth factors. The combination of skeleton and minerals contributes to the great strength and resiliency of bone tissue, providing adequate assistance and safety for routine physical activity (Wawrzyniak and Balawender, 2022). Based on blood vessel distribution and porosity, the bone may be further subdivided into the cortical bone, which is the outermost layer having poor porosity and limited blood vessels (10%), as well as bone cancellous, which is the primary element of the interior arrangement, with many blood vessels and higher porosity (50%–90%) (Mukkamalla and Malipeddi, 2021). Because of a sufficient enough supply of blood, Bone tissue has a restorative capacity and can regenerate itself through continual bone remodeling that responds to the ever-changing body burden and retain the required mechanical strength (Lee et al., 2021). Though, not every bone fracture may resolve on its own. Once injury surpasses the bone’s maximum capability for self-repair, external interference is necessary (Roddy et al., 2018). Due to their inherent limitations, conventional bone repair strategies are frequently incapable of achieving fast and efficient bone tissue restoration. For example, autografts have restricted donor access and might cause morbidity at the donor site. Moreover, allografts are vulnerable to immunological rejection and the possibility of disease transfer (Giannoudis et al., 2005). Other metallic or nonmetallic grafts are employed to rebuild the structure the role of damaged bone tissues must also contend with resistance to corrosion and attachment of bacteria (Kumar et al., 2020). Consequently, novel bone repair materials must be developed. Since bone ECM is predominantly composed of well organised nanocrystalline HA and collagen nanofibers (McMahon et al., 2013) (Figure 6). Biomimetic nanoparticles with exceptional physical and chemical characteristics have found momentum in orthopaedic clinical studies and applications (Qiao et al., 2022).

### Bone graft nano-biomaterials

The most prevalent cause of bone loss (Carrington, 2005; Jimenez-Andrade et al., 2010; Haagsma et al., 2016) are fractures, osteoporosis, and OA caused by trauma, tumors, and the aging process. A bone loss exceeding twice the diameter of the long bone diaphysis (for example, a serious bone abnormality) is not likely to restore on its own despite advancements in clinical treatment (Roddy et al., 2018). Whereas autologous bone transplantation remains the excellence standard in bone healing (Fleming et al., 2000), inadequate bone supplies and inevitable morbidity at the donor location have severely restricted its use. Over the previous few years, bone tissue engineering for the cure of bone abnormalities has developed rapidly and made significant progress. Particularly, nano-scale biological scaffolds exhibited an enhanced ability to replicate the natural ECM 3D structure, fostering the adhesion, propagation, and osteoblast differentiation, with less constraints than conventional graft healing procedures.

Bone TE may be restricted to the use of seed cells as a source of new bone growth, the use of scaffolds to aid in cell attachment and immigration, as well as the inclusion of bioactive substances that induce differentiation of osteogenic cells, which is reliable with the critical components of bone repair in the human body (Giannoudis et al., 2005). As an element of the ECM, the scaffold mediates intercellular signaling and relations (Danišovič et al., 2012). The scaffold provides the necessary structure for cell adhesion and development. Consequently, The efficacy of regenerating tissue is determined by the scaffold’s ability to replicate the makeup and structure of bone. An optimal bone tissue scaffold material must be biocompatible and biodegradable, have an appropriate porosity and size of the pore, and possess specific mechanical characteristics. In addition, it must be capable of modulating the attachment, propagation, and bone-forming differentiation of seed cells (Martins et al., 2018) via surface modification of bioactive molecules. In comparison to previous scaffolding components (ceramics, metals, and polymers), nano-biomaterials have enabled the development of biomimetic scaffolds with a related hierarchical organization to that of native bone, thereby effectively mobilizing matching cells at the bone-implant interface during bone remodeling when offering enough mechanical qualities to adapt to a range of loading scenarios.

### Nanofiber

Nanofibers (NFs) are better suited for use as scaffold constituents than NPs of their continual nature. The beneficial effects of nanofibrous scaffolds comprise their high surface-to-volume ratio, significant porosity, along with morphological resemblance to native ECM, which can recreate a biomimetic environment to influence cell-matrix relations and generate beneficial cell actions (propagation, attachment, and distinction) (Li et al., 2002). Electrospinning, separation of phases, as well as self-assembly, shared creation methods for NFs (Smith et al., 2008; Udomluck et al., 2019), with electrospinning, is the most preferred method because it eases of use and the diversity of created scaffolds. During electrospinning, an electrostatic force is provided to a voltage-applied polymeric solution (Maxwell stress) larger than its surface tension. As a result, when charged streams evacuate the polymeric solution, it is stretched several times and fractured, resulting in the creation of nanoscale fibers (Doshi and Reneker, 1995). Artificial polymers like poly (glutamic acid), poly (lactic acid), and poly (-caprolactone) (PCL) as well as natural polymers, i.e., chitosan, collagen, alginate, and gelatin (Hing, 2004), have been utilized to create NFs to date. A previous study reported that a versatile 2-spinnerette method for fabricating nanofiber scaffolds with compositional gradients has been developed. This method can be used to fabricate nanofiber gradients from any electrospinnable material. Several critical device modifications that make the approach reliable and effective were identified. The application of nanofiber composition gradients to tissue engineering was demonstrated by creating gradients. Osteoblast adhesion and proliferation were modulated by nanofiber scaffolds with nACP concentration gradients. These findings indicate that nACP nanofiber gradients can be used to engineer interface tissues containing osteoblast gradients, such as ligaments and tendons (Ramalingam et al., 2013). The mineral content was found to be positively correlated with local expressions of Runx2, ALP, and OCN, resulting in a geographical gradient of cell morphologies. This gradient of cell morphologies closely reflects the cellular population of the natural enthesis and so represents a potential initial step towards tendon-to-bone insertion rejuvenation (Liu W. et al., 2014).

### Nanofibrous scaffolds made of a single ingredient

Natural polymers are considerably less common for bone reparation while having greater biocompatibility and enriched arginine-glycine-aspartate (RGD) ingredients. This could be the result of inadequate mechanical strength and unpredictable *in vivo* deterioration. In bone tissue engineering, polymers of synthetic origin with higher physical characteristics and regulated rates of degradation are gaining popularity. After 4 weeks of active cultivation using rat MSCs, Yoshimoto et al. (2003) observed that osteoblast-like cells adhered to the PCL surface created by electrospinning. Additionally, it was determined that COL-I accumulation and matrix mineralization were acceptable, establishing the electrospun PCL matrices have the potential as a tissue bone scaffold. Though, traditional electrospinning generates compact, dense NFs with tiny surface voids. Moreover, it can only create 2D matrices which are not suitable for cell entry or transmission of signals, thereby diminishing implanted cells’ long-term viability (Rnjak-Kovacina and Weiss, 2011; Cai et al., 2012). To optimize the structure of pores (for instance size, interconnectivity, as well as porosity) of NFs, researchers have been working to enhance the fabrication procedure. Eap et al. (2012), by altering process variables (for example, feed rate and voltage) and PCL solution features (for instance conductivity and viscosity), a 3D scaffold with a diverse arrangement of porosity and macropores rating of 93 percent was produced. They revealed that a 3D framework might promote cell development *in vitro* in comparison to a 2D framework. In terms of topography/morphology, one more 3D PCL scaffold created by Xu et al. (2015) showed that porosity was increased to 96.4 percent with a maximal pore size of 300 m by thermally induced self-agglomeration (TISA) technology, more closely resembling native ECM. *In vitro* investigations indicated that the scaffolds encouraged BMP-2-induced chondrogenic growth of mouse BMSCs, which was followed by functional bone renewal using a physiological ossification of the endochondral approach. In addition to electrospinning, additional procedures have been devised for the production of NFs. Hartgerink et al. (2001) created a peptide-amphiphile scaffold with reversible NF cross-linking using a pH-controlled self-assembly technique. Peptide-amphiphile NFs should produce well-aligned HA on their surface during mineralization studies, which was very similar to the nanostructure of actual bone. Table 2 summarizes the application of various nanomedicine in the orthopedics.

**TABLE 2:** Examples of modern nanomedicine applications in orthopedics.

Applications	Medical condition	Nanocomponent	References
Drug delivery	Tendonitis refers to tendon inflammation (the fibrous tissue strands that connect muscles to bones), which causes pain, stiffness, and swelling	Autologous tenocyte injection has been shown to improve tendon remodeling, collagen content, and tensile strength. The integration of tenocytes was tracked employing nanoparamagnetic iron oxide	Chen et al. (2011)
Diagnostics	Herniated disc—occurs when an intervertebral disc degrades and the interior ruptures of the nucleus. The material parts of the herniated disc then put stress on the nerve roots, creating discomfort, weakness, numbness, or sensory alterations	Mechanical assessment of nanoscale properties of the annulus fibrosis via atomic force. The nanoscale stress/strain and hydration properties were studied. In contrast to the current mechanical prosthesis, this represents a considerable advancement in providing tissue replacements in cases of spinal damage	Deng et al. (2023)
Biomaterials	Osteoarthritis is characterized primarily by cartilage degradation and narrowing of joint spaces; It may also contain bone overgrowth, spur formation, and reduced function	The use of anodized TiO_2_ nanotube surface structures reduced nitrous oxide production and the formation of fibrotic capsules	Kingsak et al. (2022)
Therapeutics	Rheumatoid arthritis is an inflammatory disorder that affects the lining of the joints (synovium). The inflammation may affect the whole joints	Inner hollow nanospheres in chondroitin sulphate solution that have been chemically changed. The spheres will enhance traditional chondroitin sulphate therapy by adding medicine or growth strategies	Wen et al. (2023b)
Imaging	Scoliosis is characterized by lateral, or sideways, rotations and curvature of the back bones (vertebrae), giving the perception that a person is leaning to one side	Neuro-central growth plate nanostructure imaging. Abnormalities as little as a nanometer have been discovered to cause idiopathic scoliosis and other disorders of the neurocentral growth plate	Hickman et al. (2022)
Biomaterials	Osteoporosis is characterized by bone mass loss and disintegration of bone tissue. The procedure weakens the bones thereby rendering them more liable to fracture	A bone morphogenetic protein-loaded nano-HA ceramic/polymer composite (BMP-7) enables long-term drug administration and bone scaffolding	Mo et al. (2023)
Implants	Osteomyelitis (postoperative)—infection caused by contamination of the wounded region, most commonly caused by bacterial adherence to implants	Sharklet AFTM, a surface microtopography based on shark skin, has been shown to inhibit bacterial biofilm development without the use of bacteriocidal chemicals	Balanger et al. (2017)
Biomaterials	Fracture— an impact-induced partial or full fracture or break of a bone	Restoration of a radial lesion using a nano-HA bone scaffold with 100–250 lm holes. Porous nano-HA enhanced bone formation and biocompatibility	Xue et al. (2022)
Drug delivery	Osteosarcoma is serious cancer that frequently affects adolescents and teenagers	A polymeric nanosystem is utilized to give doxorubicin to osteosarcoma patients	Chen et al. (2023)
Sensor	Fracture— a partial or full fracture or crack of a bone induced by trauma	Multiwalled carbon nanotubes will be able to identify new bone formation *in vivo*. Because the electrical properties of bone differ from those of nearby tissue and fibrous scars, it is possible to evaluate the electrical conductivity of new growth to find out which tissues are growing. This data can subsequently be relayed to a radio frequency receiver located outside the body	Norizan et al. (2020)

### Prospective concerns

In the early phases of research, nanomaterials have been linked to brain and lung cytotoxicity, oxidative stress, and Inflammation throughout the body (Polyzois et al., 2012). Nonetheless, further investigation suggests that the effects of nanoparticle metabolism might improve microscopic lung and bone cell health (Sato and Webster, 2004). When sufficient grafts are already available, the amalgamation of these two challenges could deter numerous medical expedient manufacturers from spending million of dollars in capital (Nodzo et al., 2015). Since 2008, only 3 percent’s nanotechnology investigating resources have been allocated to health studies (Sullivan et al., 2014). Before nanomaterials can be utilized widely used in medicinal purposes, it will be necessary to conduct extensive research to determine their potential toxicity.

Another obstacle is the bulk production of nanomaterials. According to some specialists, it is not possible to mass-produce substances with a diameter of less than 0.3 nm because of their complex structural characteristics. Kelly and Dean (2016), revealed that the size and physical features of each component can fluctuate when these NPs are mass-produced at a relatively low level. As a consequence, it may be impossible to accomplish the raised, paradigm of low-cost manufacturing with certain NPs without sacrificing a certain degree of repetition.

### The commercialization of nanotechnology and potential challenges

Global commercialization efforts for nanomedicine have now begun. Of the 200 businesses recognized as being involved in nanomedicine throughout the world, 159 are beginning small and medium-sized businesses that specialize in pharmaceutical development and medical devices enhanced by nanotechnology. In addition, 41 prominent pharmaceutical and medical equipment firms have nanomedicine products on the market or are involved in nanotechnology-related research and development. During the previous decade, 38 medical items using nanotechnology enhancements have been presented to the market, with 2004 sales projected at EUR 5.4 billion. Despite the various hurdle in the commercialization of nanotechnology in orthopedics, we believe nanotech-based drug delivery and diagnostic approaches will soon be translated to clinics. Based on a pipeline of approximately 157 products in advanced development, the market for nanomedicine products is expected to reach approximately 15 billion euros in 2012. Currently, comparable to research activities, drug delivery systems account for about 80% of the nanomedicine market. Therapies based on nanotechnology, *in vitro* diagnostics, and imaging agents are still in the developmental phases, but it is anticipated that their significance will grow substantially in the future. Although the EU leads in terms of scientific publications, it is less competitive when it comes to commercialization: US firms are engaged in 46% of nanomedicine products on the market, whereas EU25 companies have only 35%. Considering the product pipeline, this disparity appears to grow. This is primarily due to the feeble position of EU25-based nanomedicine firms in the drug delivery sector, where they represent only 23% of firms compared to 60% of US firms. The proportion of European companies in the medication delivery sector, the nanomedicine application area with the greatest market potential at present, is roughly two times less than in the other nanomedicine application areas.

There are many obstacles to the development of nanomedicine, but one of the most significant is the pharmaceutical and medical device industries’ still-modest interest in this new technology. Entrepreneurs are presently pursuing a plethora of nanotechnology-based suggestions for better illness diagnosis and care, however, they are having difficulty finding significant pharmaceutical or healthcare device companies that will license their technology or collaborate with them to gain regulatory consent for their new nanomedicine techniques. This case is not wholly novel to the medical industry, as it recalls the development of biotechnology-based medications over the last three decades. In addition to a lack of interest on the part of large pharmaceutical companies, experts warn that there is a fundamental cause for the sluggish pace of nanotechnology adoption in the healthcare profession, mainly in Europe: Experts view the cost-regulated markets of main EU25 nations as a significant barrier to the advancement of inventive, high-value pharmaceuticals, comprising nanomedicine. In addition, the vast majority of the therapeutic and diagnostic benefits of nanotechnology-based medications and contrast agents will stem from their capacity to target illnesses more precisely and provide more precise diagnostic data. This results in more limited patient groups and, consequently, a smaller market for nanoparticle products, making it more challenging to recoup growth and the expenses of regulatory approval and potentially rendering an economically undesirable development.

Substantial investigative attempts have been expended over time to design and modulate the biomaterial features to acquire an application-specific biological reaction (Huebsch and Mooney, 2009; Achterberg et al., 2014). For example, the hardness of the cell substrate may be adjusted to maximize muscle cell growth (Griffin et al., 2004). Orthopedic biomaterials are classified into 02 types in the modern context: nanophase and traditional biomaterials. Traditional biomaterials can be subdivided into 1) metals and metal alloys and 2) substances that are not metallic. (For example, polymeric, carbon composites, crystalline ceramics, and amorphous glasses). This section contains, conventional biomaterials and their attendant challenges for orthopaedic implant applications are discussed.

## Future perspectives and final remarks

Nanobiomaterials can be used in orthopedics, according to preliminary research; however, additional improvements are necessary to achieve practical application. The aim is to generate functional bone-repairing matrices that can restore natural tissues partly while interacting in relation to their surroundings, reacting to environmental variations, and actively influencing cellular processes to speed up bone creation, shorter healing times, and a quicker recovery to function. Future investigation will almost definitely concentrate on enhancing design methods employing NPs and other manufacturing methods. It is crucial to comprehend the molecular mechanisms underpinning cell-nanobiomaterial connections. In addition, caution should be taken when proving nanomaterial biosafety and limiting their consequences. Concerns exist regarding the toxicity of NPs produced by wear and strain. At the level of the nanoscale, metals behave differently and exhibit different material characteristics than at the microscale.

Therefore, conventional implants with specific features treated using nanotechnology are preferred to transplants composed of nanoparticles. This precludes the possibility of nanomaterials disseminating and influencing tissue toxicity. Given these reservations, Regulation has been proposed as a required step. Companies are still reticent to craft nanostructured implants and artificial limbs, because of their uncertain medicinal/therapeutic advantages, probable harmfulness, and expensive price (Smith et al., 2018), which are some main concerns about the toxicity of NPs produced by the use and strain. Metals react differentially and have various material characteristics at the level of the nanoscale than they do at the microscale.

Decellularized extracellular matrix (dECM) generation is a revolutionary strategy for creating biomimetic biomaterials. Decellularization is the process of successfully removing RNA, DNA, and additional components derived from tissues or organs whereas preserving the extracellular matrix’s (ECM) original composition and structural integrity (Pati et al., 2015; Zhang et al., 2016), thereby attaining the objective of reducing rejection of allografts and xenografts’ cellular antigen-induced responses. In addition to being less immunogenic, the decellularized ECM components nevertheless include a sizeable portion of the native ECM’s active components (for instance GAGs, collagen, and growth factors), which assemble themselves into gels and can be used as injected matrices for tissue restoration and healing, availability of seed cells as well as developmental materials (Hoshiba et al., 2010; Taylor et al., 2018). It is been established that employing homologous dECM scaffolds to restore matching tissues can stimulate regeneration of tissues and healing further efficiently as compared to scaffolds produced from various sources of material (Sawkins et al., 2013). Therefore, the most efficient organic substance for chondral/bone tissue rejuvenation is without a doubt dECM made from chondral/bone tissue. Pati et al. (2014), employed a mixture of bone tissues PCL and dECM to produce bone skeletons with great mechanical strength as well as stability. Similarly Beck et al. (2016a), developed decellularized cartilage gels that were methacrylated and had elastic compressive moduli comparable to natural cartilage in particular regions (1,070–150 kPa). Furthermore, to structural and chemical resemblances with natural ECM, dECM promotes the parameters in the host like stem cell growth and differentiation (Luo et al., 2015). Luo et al. injected stem cells from a human infrapatellar adipose pad onto a scaffold made of cartilage’s extracellular matrix and discovered improved cell proliferation. Choi et al. (2021), investigated in separate research the efficacy of a bone dECM-implanted electrospun PCL-based fibrous scaffold in comparison to PCL, the outcomes demonstrated that dECM had no influence on the physical qualities of the matrices, but had a considerable impact on cell adhesion, propagation, and differentiation. Lu et al. (2021) contrasted the distinctions concerning two collagen matrix approaches (in solution and solid form, correspondingly) enhanced with porcine dECM. Even though both types of dECM facilitated MSC enrollment, propagation, and chondrogenic differentiation, the latter group performed better, demonstrating a unique distinction between the two types of scaffolds in the regional microenvironment of cells formed by the dynamic modulation of biological variables throughout time, indicating that the manufacturing methods must be optimized when developing dECM-based scaffolds. In conclusion, dECM demonstrates a distinctive bioactive natural scaffold material that may be used to construct bone and cartilage tissue. Following full vs. moderately fragmented decellularization therapy, the biological activity of the dECM generated does not significantly differ from each other (Keane et al., 2012); though, few research studies have indicated that severe decellularization therapy can reduce ECM’s biological activity. Moreover, current decellularization procedures have inevitable deleterious consequences on ECM, needing further advancement of dECM physical, biological, and structural qualities throughout future formulation (Beck et al., 2016a; Beck et al., 2016b). In addition, it is difficult to increase the dECM reproducibility volumes while simultaneously scaling up manufacturing.

The future of cartilage and bone restoration would be gene-based therapy because it focuses on the processes involved in a certain illness, addressing the underlying causes instead of focusing on the symptoms. To attain this objective, a secure and efficient distribution approach is required. Although the great transfecting effectiveness of viral carriers is utilized in most pharmacogenomic approaches, its common limitations/restrictions, including immune and cellular toxicity (Ramamoorth and Narvekar, 2015), have a significant effect on clinical translation success. Current improvements in nanomedicine have led to the advancement of diverse non-viral vectors of genes that are in various stages of clinical trials. Examples include the manufacturing of peptide-NF-B siRNA NPs by Yan et al. (2016) proven to freely enter bone tissues and remain active for >14 days, thereby boosting AMPK signaling while reducing mTORC1 and Wnt/-catenin activity hence preserving cartilage integrity. PLGA-based NPs have also been utilized in cartilage injury gene therapy. Shi et al. (2013), fruitfully transfect BMP-4 plasmids into rabbit ADSCs while using PLGA NPs Additional research revealed that this nanocomplex may efficiently stimulate chondrogenesis *in vivo* and *in vitro* in matrices implanted with it. Furthermore, a comparatively novel siRNA delivery system class, e.g., liposomal nanoparticles, is anticipated to be a potent instrument for curing bone injury via silencing precise genes (Wang et al., 2018). Liposomal NPs are capable of transfecting 100 percent of chondrocytes.

Despite the emergence of nanomaterial-based therapeutic products in orthopedic disciplines, for example, drug distribution, biosensors, and scaffolds made of tissue-engineered materials, sample amounts in relevant research are small, as well as long-term planning is inadequate. Nanotoxicity and responses to inflammation cannot be disregarded and need additional preclinical research to ensure safety, given that previous research has focused on enhancing the quality of graft-host assimilation using diverse materials. It is crucial to improve communication between doctors and lab staff to provide solutions to a variety of problems, as well as constant improvement the fit between nano-design and production procedures (for instance, expanding the manufacturing of nanomaterials well-suited with 3D printers and integrating computer-assisted design in combination with analysis of finite elements to better comprehend the connection between mechanical characteristics and scaffold structure).

Even though nanotechnology is still in its infancy, It can enhance orthopedic diagnosis, management, and research. The performance of commercial and service industries verifies the notion that nanotechnology will play a crucial role in future treatment methods. Nanotechnology with the potential to significantly lower the budget of a handful of traditional medications and with the facilitation of an abundance of new applications. Nanotechnology makes accurate methods for therapy methods, as a result of which more efficient and durable grafts, reduced infection prevention, and improved bone and tendon renewal. Massive efforts in fundamental science research are beginning to realize the potential benefits of nanomedicine, particularly in orthopedics. Though, further study is required to fully understand the safety and utility of this new technology.
